# Supplementary material for: Metabolomic and Proteomic Signatures of Cardiorespiratory Fitness for Predicting All-Cause Mortality and Non-Communicable Disease Risk: A Prospective Study in the UK Biobank
Source: Circ Genom Precis Med. 2026 Jul 3;19(4):e005736. doi: 10.1161/CIRCGEN.125.005736 (PMC13489766; doi:10.1161/CIRCGEN.125.005736)
Supplement: Supplementary file 1 [file hcg-19-e005736-s001.pdf]

SUPPLEMENTAL MATERIAL

Supplemental Tables

Table S1. Definition of outcome, exposure and covariates.

**Exposure**	**Definition and source**
**Cardiorespiratory fitness (CRF)**	VO_2max per kg bodyweight estimated from the exercise test (ID: 30038). This estimation was obtained from heart-rate monitoring (using a 4-lead electrocardiograph [ECG]; CAM-USB 6.5, Cardiosoft v6.51) during cycle ergometry on a stationary bike (eBike, Firmware v1.7) with a ramp slope that is adapted to the individual based on age, gender, weight and medical history. ECGs were recorded pre-test (15 seconds), during activity (6 minutes) and in recovery (1 minute). The participant's risk category was first calculated to determine what level of activity they should perform or have only a resting ECG performed. The percentage levels of effort during activity were then determined according to their risk category, and their maximum workload was calculated according to age, height, weight, resting heart rate and sex.
**Metabolomic profiles**	NMR metabolic biomarkers (ID: 220): NMR metabolic biomarkers generated by Nightingale Health. Metabolic biomarkers were measured from randomly selected EDTA plasma samples using a high-throughput NMR-based metabolic biomarker profiling platform developed by Nightingale Health Ltd. The measurements took place between June 2019 and April 2020 (Phase 1) and April 2020 and June 2022 (Phase 2) using eight spectrometers at Nightingale Health, based in Finland. The biomarkers span multiple metabolic pathways, including lipoprotein lipids in 14 subclasses, fatty acids and fatty acid compositions, as well as various low-molecular weight metabolites, such as amino acids, ketone bodies, and glycolysis metabolites quantified in molar concentration units.
**Proteomic profiles**	Protein biomarkers (ID: 30900): Protein biomarkers were measured in EDTA plasma samples from approximately 54,000 UK Biobank participants using the Olink® Explore 3072 platform, a high-throughput multiplex immunoassay based on proximity extension assay (PEA) with next-generation sequencing (NGS) readout. Protein expression levels are reported in Normalized Protein eXpression (NPX) units on a log2 scale, enabling relative quantification across samples.
**Outcome**	**Definition and source**
**All cause mortality**	Ascertained from Office for National Statistics death records.
**Cardiovascular disease (CVD)**	Any of the following:<br>- Reporting diagnosis of heart attack, angina, or stroke by a physician. (Touchscreen or verbal interviews)<br>- HES: total CVD was defined as a hospital admission/episode or death with ICD-10 codes including coronary heart disease (I20-I25), congestive heart

	failure or cardiomyopathy (I50, I50.1, I50.9, I11.0, I13.0, I13.2, I42, I43.1), and total stroke (I60-I64).<br>- Fatal CVD events were defined by I00–I25, I27–I88, and I95–I99 in any of the causes of death in the ONS death record.
**Cancer**	Cancer diagnosis and mortality were obtained from the UK Biobank cancer registry using ICD-10 codes for lung cancer (C34), breast cancer (C50), colon cancer (C18), colorectal cancer (C18–C20), bladder cancer (C67), prostate cancer (C61), and endometrial cancer (C54.1). For each type, the earliest diagnosis date was extracted from field 40005 if a matching ICD-10 code was present in field 40006. Diagnoses were then classified as incident (occurring after the baseline assessment date; field 53-0.0) or prevalent (on or before baseline). Cancer-specific mortality was identified using primary (field 40001) and secondary (field 40002) causes of death, with the date of death obtained from field 40000.
**Type 2 diabetes**	HES: hospital admission/episode with ICD-10 codes for non-insulin dependent diabetes (E11.0-E11.9), malnutrition-related diabetes (E12.0-E12.9), other specified diabetes (E13.0-E13.9), unspecified diabetes (E14.0-E14.9), pre-pregnancy type 1 diabetes (O24.0), pre-pregnancy type 2 diabetes (O24.1), pre-pregnancy malnutrition-related diabetes (O24.2), and pre-pregnancy unspecified diabetes (O24.3)
**Covariates**	UK Biobank variable (ID), source and time point (where relevant)
**Age**	Age at recruitment (ID: 21022)
**Ethnicity**	Ethnic background (ID: 21000) – most recent time point available
**Townsend index**	Townsend index (ID: 189)
**Education**	Qualifications (ID: 6138) – highest qualification reported among the time points of assessment.
**Family history of diabetes**	Family history of diabetes (ID: 20107, 20110, 20111) - present if reported in at least one time point.
**Family history of CVD**	Family history of CVD (ID: 20107, 20110, 20111) - present if reported in at least one time point.
**Family history of cancer**	Family history of cancer (ID: 20107, 20110, 20111) - present if reported in at least one time point.
**Geographical and location**	Assessment Centre (ID: 10)
**Body mass index (BMI)**	BMI (ID: 21001) – average from available time points

**Menopause**	Had menopause (ID: 2724) - present if reported in at least one time point
**Smoking**	Smoking status (ID: 20116) – latest time point available
**Physical activity**	Number of days/week of vigorous physical activity 10+ minutes (ID: 904) – average from available time points; Duration of vigorous activity (ID: 914) – average from available time points; Number of days/week of moderate physical activity 10+ minutes (ID: 884) – average from available time points; Duration of moderate activity (ID: 894) – average from available time points; Number of days/week walked 10+ minutes (ID: 864) –average from available time points; Duration of walks (ID: 874) – average from available time points.
**Fruit and vegetable intake**	Sum of:<br>- Cooked vegetable portions per day (ID: 1289) – average from available time points.<br>- Raw vegetable portions per day (ID: 1299) – average from available time points.<br>- Fruit portions per day (ID: 1309) – average from available time points.
**Alcohol intake**	Alcohol weekly intake (units/week) – sum of:<br>- Weekly red wine intake (ID: 1568) – amount of glasses per week multiplied by 2, and taking the average from available time points .<br>-Weekly beer and cider intake (ID: 1588) – amount of pints per week multiplied by 2.5, and taking the average from available time points.<br>- Weekly champagne and white wine intake (ID: 1578)– amount of glasses per week multiplied by 2, and taking the average from available time points.<br>- Weekly spirits intake (ID: 1598) – amount of measures per week multiplied by 1, and taking the average from available time points.<br>- Weekly fortified wine intake (ID: 1608) – amount of glasses per week multiplied by 2, and taking the average from available time points.<br>- Weekly other alcoholic drinks intake (ID: 5364) – amount of glasses per week multiplied by 1, and taking the average from available time points.<br>- Monthly red wine intake (ID: 4407) – amount of glasses per month multiplied by 2 and divided by 4.3, and taking the average from available time points.<br>- Monthly beer and cider intake (ID: 4429) – amount of pints per month multiplied by 2.5 and divided by 4.3, and taking the average from available time points.<br>- Monthly champagne and white wine intake (ID: 4418) – amount of glasses per month multiplied by 2 and divided by 4.3, and taking the average from available time points.<br>- Monthly spirits intake (ID: 4440) – amount of measures per month multiplied by 1 and divided by 4.3, and taking the average from available time points<br>- Monthly fortified wine intake (ID: 4451) – amount of glasses per month multiplied by 2 and divided by 4.3, and taking the average from available time points.<br>- Monthly other alcoholic drinks intake (ID: 5364)– amount of glasses per

	month multiplied by 1 and divided by 4.3, and taking the average from available time points.

Table S2: Characteristics of participants included in the metabolomic and proteomic validation cohorts.

		METABOLOMIC VALIDATION COHORT	PROTEOMIC VALIDATION COHORT
**Characteristic**	**Category**	**Frequency of participants (% full cohort)**	**Frequency of participants (% full cohort)**
Full-cohort		354,222 (100.0%)	29,961 (100.0%)
Age (years)	39-44 yrs	41,445 (11.7%)	3,446 (11.5%)
	45-49 yrs	51,622 (14.6%)	4,181 (14.0%)
	50-54 yrs	57,279 (16.2%)	4,706 (15.7%)
	55-59 yrs	65,419 (18.5%)	5,453 (18.2%)
	60-64 yrs	80,975 (22.9%)	6,839 (22.8%)
	65-69 yrs	56,129 (15.8%)	5,186 (17.3%)
	≥70 yrs	1,351 (0.4%)	150 (0.5%)
	Unknown	2 (<0.1%)	0 (0.0%)
Sex	Female	198,873 (56.1%)	16,797 (56.1%)
	Male	155,349 (43.9%)	13,164 (43.9%)
Body mass index ($kg \cdot m^{-2}$)	<18,5	1,971 (0.6%)	173 (0.6%)
	18.6-24.9	122,361 (34.5%)	10,278 (34.3%)
	25-29.9	151,273 (42.7%)	12,888 (43.0%)
	30-34.9	57,153 (16.1%)	4,781 (16.0%)
	35-39.9	14,845 (4.2%)	1,236 (4.1%)
	40≤	5,231 (1.5%)	471 (1.6%)
	Unknown	1,388 (0.4%)	134 (0.4%)
Ethnic background	White	336,905 (95.1%)	28,189 (94.1%)
	Black	4,624 (1.3%)	614 (2.0%)
	Asian	6,450 (1.8%)	522 (1.7%)
	Mixed	4,748 (1.3%)	496 (1.7%)
	Unknown	1,495 (0.4%)	140 (0.5%)
Townsend deprivation index	Least deprived	74,940 (21.2%)	6,237 (20.8%)
	Less deprived	72,646 (20.5%)	6,048 (20.2%)
	Moderately deprived	71,855 (20.3%)	5,724 (19.1%)
	More deprived	68,887 (19.4%)	5,961 (19.9%)
	Most deprived	65,469 (18.5%)	5,954 (19.9%)
	Unknown	425 (0.1%)	37 (0.1%)
Qualifications	Higher education	169,251 (47.8%)	14,289 (47.6%)
	Secondary education	104,028 (29.4%)	8,719 (29.1%)
	Vocational education	20,173 (5.7%)	1,699 (5.7%)
	None of the above	55,152 (15.6%)	4,812 (16.1%)
	Unknown	5,618 (1.6%)	442 (1.5%)
Smoking status	Never	198,981 (56.2%)	16,690 (55.8%)
	Previous	116,470 (32.9%)	9,928 (33.1%)
	Current	37,211 (10.5%)	3,213 (10.7%)
	Unknown	1,560 (0.4%)	130 (0.4%)
Alcohol intake	Never	25,489 (7.2%)	2,356 (7.9%)
	Occasional	77,572 (21.9%)	6,597 (22.0%)
	Moderate	177,616 (50.1%)	14,825 (49.5%)
	High intake	72,877 (20.6%)	6,124 (20.4%)
	Unknown	668 (0.2%)	59 (0.2%)
Meet physical activity recommendations	Yes	223,034 (63.0%)	18,834 (62.9%)
	No	50,670 (14.3%)	4,294 (14.3%)
	Unknown	80,518 (22.7%)	6,833 (22.8%)
History of menopause	Yes	116,868 (33.0%)	10,022 (33.5%)
	No	81,822 (23.1%)	6,754 (22.5%)
	Unknown	155,532 (43.9%)	13,185 (44.0%)
Geographical region	East Midlands	27,885 (7.9%)	2,758 (9.2%)
	London	32,300 (9.1%)	3,272 (10.9%)
	North East	47,043 (13.3%)	3,851 (12.9%)
	North West	58,286 (16.5%)	4,792 (16.0%)
	Scotland	28,928 (8.2%)	2,514 (8.4%)
	South East	36,903 (10.4%)	2,506 (8.4%)
	South West	36,307 (10.2%)	2,681 (8.9%)
	Wales	16,919 (4.8%)	1,483 (4.9%)
	West Midlands	22,936 (6.5%)	1,850 (6.2%)
	York	46,715 (13.2%)	4,254 (14.2%)

Meet recommended fruit and vegetable intake	Yes	267,066 (75.4%)	22,632 (75.5%)
	No	79,762 (22.5%)	6,619 (22.1%)
	Unknown	7,394 (2.1%)	710 (2.4%)
Family history of cardiovascular disease	Yes	258,262 (72.9%)	21,827 (72.8%)
	No	90,457 (25.5%)	7,658 (25.6%)
	Unknown	5,503 (1.6%)	476 (1.6%)
Family history of diabetes mellitus	Yes	71,021 (20.0%)	5,954 (19.9%)
	No	277,698 (78.4%)	23,531 (78.5%)
	Unknown	5,503 (1.6%)	476 (1.6%)
Family history of cancer	Yes	13,226 (3.7%)	10,229 (34.1%)
	No	339,868 (95.9%)	19,256 (64.3%)
	Unknown	1,128 (0.3%)	476 (1.6%)

Table S3-S13 are in extended material in the excel file

Table S3. Results of linear models assessing associations between 251 circulating metabolites and cardiorespiratory fitness in the UK Biobank metabolomic discovery cohort

Table S4. Metabolites retained in the elastic net model used to derive the metabolomic signature of cardiorespiratory fitness (CRF) in the UK Biobank discovery cohort

Table S5. Metabolomics pathways using effect size obtained in the elastic net model in the discovery cohort

Table S6. Results of multivariable linear models assessing associations between 2,922 circulating proteins and cardiorespiratory fitness (CRF) in the UK Biobank proteomic discovery cohort

Table S7. Enriched biological pathways identified from proteins significantly associated with cardiorespiratory fitness in the UK Biobank proteomic discovery cohort

Table S8. Proteins retained in the elastic net model used to derive the proteomic signature of cardiorespiratory fitness (CRF) in the UK Biobank discovery cohort

Table S9. Hazard ratios by cardiorespiratory fitness quintiles for all-cause mortality and non-communicable disease risk in the discovery cohort.

Table S10. Hazard ratios by the metabolomic signature of CRF quintiles for all-cause mortality and non-communicable disease risk in the discovery cohort.

Table S11. Hazard ratios by the metabolomic signature of CRF quintiles for all-cause mortality and non-communicable disease risk in the validation cohort.

Table S12. Hazard ratios by the proteomic signature of CRF quintiles for all-cause mortality and non-communicable disease risk in the discovery cohort.

Table S13. Hazard ratios by the proteomic signature of CRF quintiles for all-cause mortality and non-communicable disease risk in the validation cohort.

Supplemental Figures

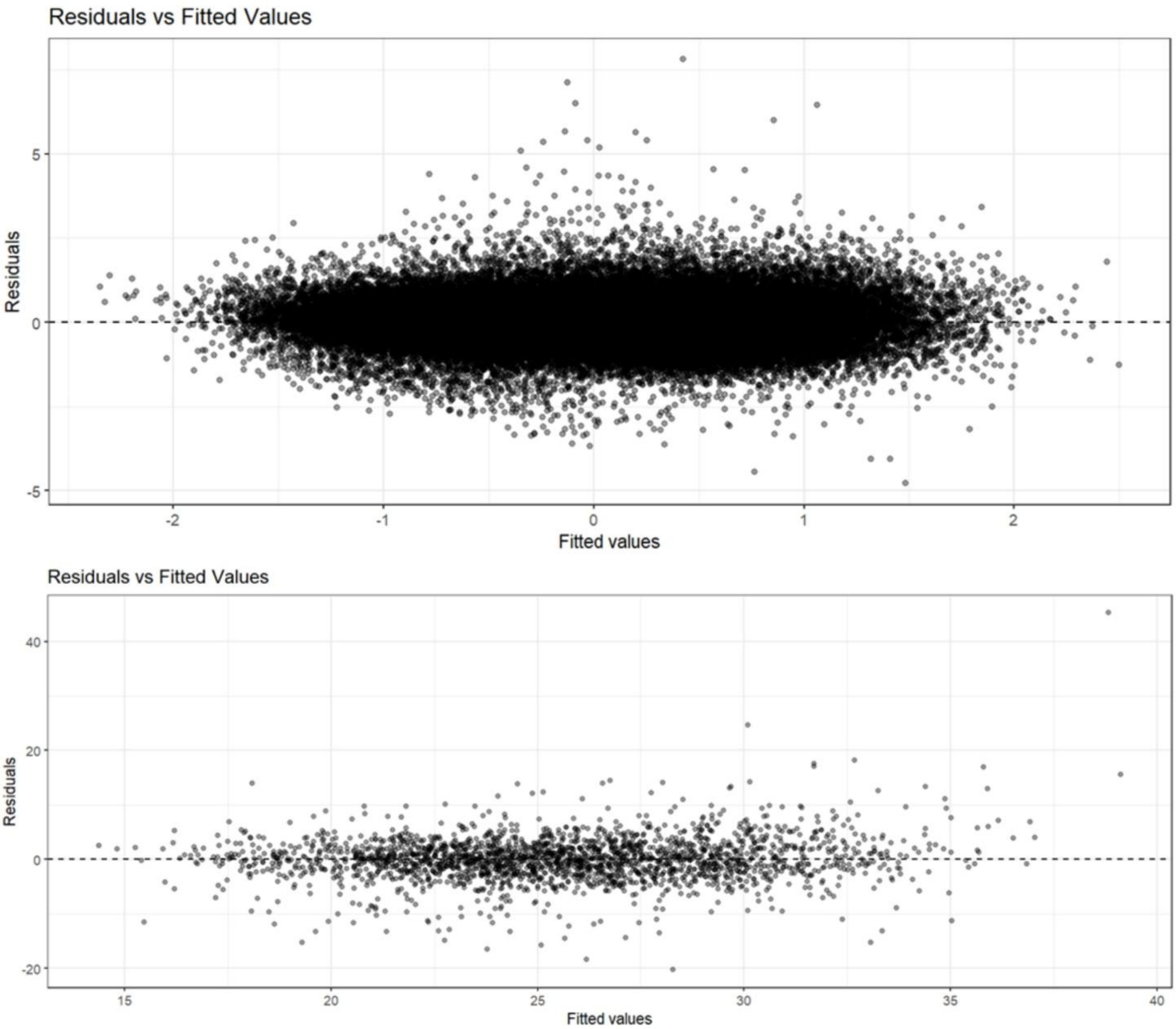

Figure S1. Residuals versus fitted values plots for calibration assessment of the elastic-net models, with the metabolomics model shown in the upper panel and the proteomics model in the lower panel.

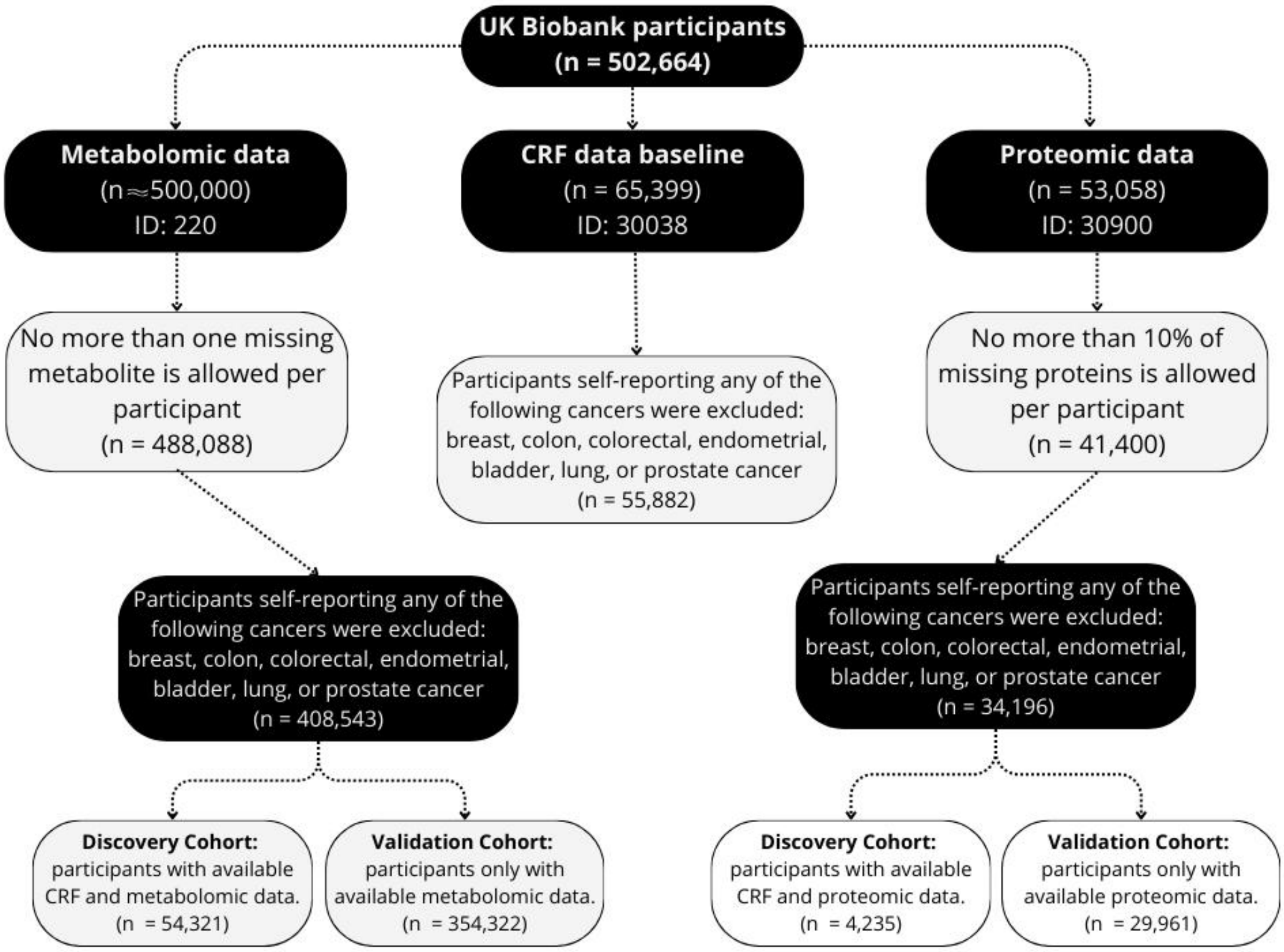

Figure S2. Participant Flow-Chart
